# Eye-mounting goggles to bridge the gap between benchtop experiments and in vivo robotic eye surgery

**DOI:** 10.1038/s41598-023-42561-9

**Published:** 2023-09-19

**Authors:** Nicholas R. Posselli, Paul S. Bernstein, Jake J. Abbott

**Affiliations:** 1 Robotics Center and Department of Mechanical Engineering, University of Utah, Salt Lake City, UT USA; 2 Department of Ophthalmology and Visual Sciences, Moran Eye Center, University of Utah, Salt Lake City, UT USA

**Keywords:** Mechanical engineering, Biomedical engineering, Preclinical research

## Abstract

A variety of robot-assisted surgical systems have been proposed to improve the precision of eye surgery. Evaluation of these systems has typically relied on benchtop experiments with artificial or enucleated eyes. However, this does not properly account for the types of head motion that are common among patients undergoing eye surgery, which a clinical robotic system will encounter. In vivo experiments are clinically realistic, but they are risky and thus require the robotic system to be at a sufficiently mature state of development. In this paper, we describe a low-cost device that enables an artificial or enucleated eye to be mounted to standard swim goggles worn by a human volunteer to enable more realistic evaluation of eye-surgery robots after benchtop studies and prior to in vivo studies. The mounted eye can rotate about its center, with a rotational stiffness matching that of an anesthetized patient’s eye. We describe surgeon feedback and technical analyses to verify that various aspects of the design are sufficient for simulating a patient’s eye during surgery.

## Introduction

A variety of robot-assisted surgical systems have been proposed to improve the precision of eye surgery^1,2^. However, to date, little attention has been paid to patient head motion, which is frequent among patients undergoing eye surgery under monitored anesthesia^3^, also known as conscious sedation, which makes a patient calm and somewhat sleepy but still often awake. Head motion in this state is due to factors such as breathing, talking, snoring, and other voluntary and involuntary motions of the patient. Brogan et al.^4^ measured the motion of 12 patients’ heads during cataract surgery and found that, over the course of a procedure, head drift was 2–7 mm medially, 2–4 mm laterally, 1–5 mm superiorly, and 1–4 mm inferiorly. In one robot-assisted in vivo study in humans, there was difficulty in initiating subretinal injection in a patient due to head drift^5^. Additionally, 16% of patients snore under monitored anesthesia, and half of those have sudden head movement during surgery^6^. Sudden movements can be unpredictable in general. Movement must be compensated for by the surgeon, to the best of their ability, to avoid complications.

Any clinically deployed robotic system for eye surgery will have to cope with patient motion. Active compensation (i.e., closed-loop control) can involve sensing the force between the surgical instrument and the eye^7^, using visual-servoing techniques^8^, or moving the headrest to counteract patient movement^9^. Others have pursued passive approaches to motion compensation, which are not mutually exclusive with active approaches. Some passive approaches include immobilizing the patient’s head^10–12^, mounting the robot to the patient’s head^13^, and forming a mechanical connection between the robot and the eye^11,14,15^.

Benchtop experiments with artificial or enucleated (i.e., ex vivo) eyes, which are typical in the development of robotic systems, rarely capture the effect of patient motion. Patient motion was simulated using a linear piezoelectric actuator that generated one-dimensional step motions by Ebrahimi et al.^7^. The robotic device proposed by Natalius et al.^9^ could potentially simulate patient motion (although it was not the proposed application), since it was designed to have the range of motion to accommodate for the patient head motion measured by Brogan et al.^4^. Any method of simulating patient motion will provide a more challenging evaluation of a robotic system than using a stationary eye, although artificially generating truly accurate physiological motion is challenging. In addition, the performance of head-mounted-robot systems and head-immobilization systems is dependent on the geometry of the patient’s head and the compliance of the soft tissues, which will not be captured by a human-head replica (e.g., a polystyrene-foam head) or a live animal (e.g., a pig). In vivo studies are more clinically realistic, but require the robotic system to be at a mature state of development, as evidenced by the relatively small number of groups conducting in vivo studies^5,15–17^.

Another aspect of eye surgery that is often neglected when robotic systems are evaluated using artificial or enucleated eyes is the tendency of eyes to rotate due to applied forces. During vitreoretinal surgery, trocar cannulas are embedded into the sclera, which act as entryways for surgical instruments. Forces exerted on the cannulas by surgical instruments may cause the eye to rotate in its orbit. Intentional rotation of the eye enables the surgeon to view different parts of the retina under the surgical microscope, but unintentional rotation of the eye due to inadvertent forces may negatively impact surgical precision. By neglecting to incorporate a way for an eye to rotate during the evaluation of a surgical robot, the eye will remain in the same orientation, whether or not there are inadvertent forces exerted on the cannulas by the surgical instruments. This may result in unrealistically high performance measures on evaluation tasks.

Furthermore, the extraocular muscles and other tissues surrounding the human eye result in elastic behavior when the eye is rotated. During eye surgery, if the eye is rotated away from its primary (i.e., relaxed) orientation, it experiences a restoring torque that tends to return it back to its primary orientation. An eye model that incorporates this elastic behavior will more realistically simulate eye surgery. The relationship between restoring torque and orientation, which can be approximated as a constant rotational stiffness^18^, may be particularly important for robotic systems using force-sensing instruments; in a clinically realistic experiment, the forces between the trocar cannulas and the surgical instruments vary based on the orientation of the eye.

Several groups have developed devices that enable artificial or enucleated eyes to be rotated. Some of these devices involve an artificial eye that rests in a bowl-shaped depression or socket^19–21^. These artificial eyes can be rotated, but they do not simulate the rotational stiffness of the eye. Two groups have created gimbal mechanisms to simulate eye rotation, but there is no mention of rotational stiffness^22,23^. Mohammadi et al.^24^ developed an eye-fixation device for surgical practice on enucleated eyes that enables the eye to rotate about its center. This system uses a vacuum pump to hold the eye in place inside of a cup. The cup rests on a low-friction surface, which enables it to be rotated easily. A weight is suspended from the cup, creating a pendulum, which simulates the rotational stiffness of an eye.

**Figure 1:**
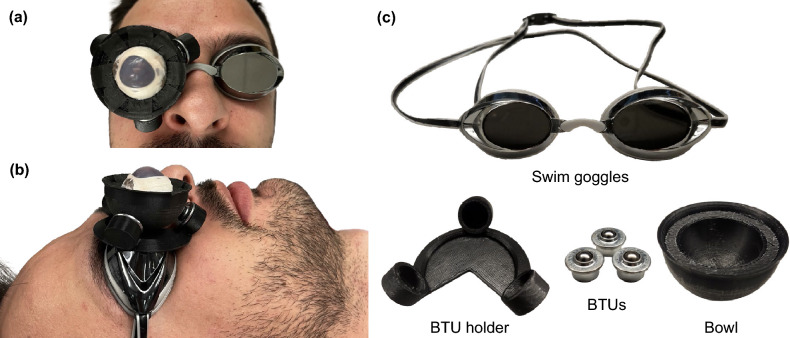
Eye-mounting goggles holding an enucleated (pig) eye. (**a**) Top-down view. (**b**) Side view. (**c**) Components used in the construction of the eye-mounting goggles. *BTU* ball transfer unit.

In this paper, we propose a device that mounts an artificial or enucleated eye to a set of standard swim goggles, which can be worn by a human volunteer during practice with, and evaluation of, eye-surgery robots (Fig. 1). Swim goggles are already designed to be comfortable and form a semi-rigid connection to the head of the wearer, and they also protect the volunteer’s eyes. Three rollers are attached to the goggles in a tripod configuration, and a bowl holding an artificial or enucleated eye is supported by the rollers. The bowl was designed using a pendulum model, which enables the eye to be rotated with a rotational stiffness that closely approximates the elastic behavior of an anesthetized patient’s eye. By mounting an eye to a volunteer’s head in this way, their head motions will be taken into account in the evaluation of a robotic system. Furthermore, the geometry of the volunteer’s head and the compliance of the soft tissues surrounding the skull will afford a more realistic evaluation of robotic systems that use head-immobilization or head-mounting methods. This concept is premised on the assumption that any robot being used has been sufficiently tested prior to the involvement of volunteers. There is a drastic gap between the types of evaluations typically conducted with eye-surgery robots (which largely involve stationary eyes) and the use of a robot in in vivo clinical trials. Our concept is intended to bridge this gap by providing a more realistic and challenging evaluation of eye-surgery robots between the initial benchtop-testing stage and the in vivo stage. Experiments where a surgical robot operates on a head-mounted eye should be approved by an ethics committee (e.g., an Independent Ethics Committee (IEC) or Institutional Review Board (IRB)). It is unrealistic that volunteers would ever be sedated to generate fully realistic patient motion. However, fully conscious volunteers can be instructed to exhibit various behaviors such as gentle breathing, deep breathing, talking, fidgeting, and even fake snoring.

Our device is similar to that of Mohammadi et al.^24^ in that it uses a pendulum design to simulate the rotational stiffness of an eye. However, our device does not require a mass to hang below the bowl, which makes our device compact enough to be mounted to a set of goggles. In Mohammadi et al.^24^, neither the magnitude of the device’s rotational stiffness nor its relationship to that of a human eye is discussed. We review literature on the rotational stiffness of an anesthetized human’s eye, and present the design process for our device to achieve this stiffness. Finally, using a vacuum pump to hold the eye in place, which could potentially distort the shape of the eye, is not necessary with our device. We use adhesive methods to attach the posterior surface of the eye to our device. The features and limitations of existing eye-mounting devices, as well as those of our proposed device, are provided in Table 1; we exclusively list devices that enable the eye to be rotated.

**Table 1:** Features and limitations of phantom/enuclated-eye testbeds that enable eye rotation.

Device	Simulates patient motion	Has rotational stiffness	Compact enough for head mounting
University of Chile device^22^	No	No	No
Tehran University of Medical Sciences device^24^	No	Yes	No
JHU eye phantom^19^	No	No	Maybe
Vanderbilt eye phantom^20^	No	No	Maybe
KU Leuven device^23^	No	No	No
JHU eye phantom^21^	No	No	No
JHU eye phantom with linear stage^7^	Yes, in 1-DOF	No	No
Our proposed eye-mounting goggles	Yes	Yes	Yes

Although our primary motivation is improved evaluation of robotic eye-surgery systems, many of the benefits of our proposed device also apply to manual practice in wet labs. Many eye-fixation devices have been developed for this application (e.g., those of Arentsen and Duran^22^ and Mohammadi et al.^24^), but they do not simulate patient motion. Lack of eye motion might make it more difficult to translate skills obtained during training to performance in clinical practice.

## Results

### Design concept

Our fabricated eye-mounting goggles, along with the individual components, are shown in Fig. 1. A solid 3D-printed polylactic-acid (PLA) bowl holds an artificial or enucleated eye. The bowl rests on three ball transfer units (BTUs), which are inserted into a 3D-printed-PLA holder. Because we are using three BTUs, the design will be robust in the sense that the weight of the bowl and eye will cause the bowl to rest on three points, and the bowl will always rotate about the center of its spherical surface. When the bowl is rotated to its maximum rotation angle, a brim around the edge of the bowl makes contact with one or more of the BTUs, acting as a hard stop to prevent the bowl from losing contact with the BTUs. The bottom surface of the BTU holder is fixed to one of the lenses in a pair of standard swim goggles using a cyanoacrlyate adhesive. To prevent the eye from moving in the bowl, masking tape is placed on the inside surface of the bowl and a cyanoacrylate adhesive is then used to adhere the posterior surface of the eye to the tape. By peeling off the tape, the eye and adhesive can be easily removed from the bowl so that a new eye may be used.

Several factors were considered in the design of the device:

- The inner diameter of the bowl should be large enough to accommodate the vast majority of human and pig eyes.
- The relationship between applied torque and rotational displacement of the eye should mimic the elastic properties of human eye rotation during surgery.
- The device should be compact, because the larger the offset from the volunteer’s eye to the mounted eye, the larger the discrepancy will be between the motion of the mounted eye and the eye of the volunteer.
- The bowl, while holding the eye, should have a high-enough weight to prevent the bowl from unintentionally being lifted off of the BTUs while it is being manipulated by surgical instruments.
- The eye should be able to rotate at least 30$^{\circ }$ from its primary orientation to approximate the range of eye rotations commonly achieved during vitreoretinal surgery.

The following sections describe how our design simultaneously achieves all of these design constraints.

### Choosing bowl inner radius to accommodate enucleated eyes

To ensure that the inner diameter of the bowl would be large enough to accommodate the vast majority of human and pig eyes, we reviewed literature that provides eye measurements for different species.

For human eyes, Augusteyn et al.^25^ reported means and standard deviations (reported in the form $\mu \pm \sigma$) of $24.44\pm 1.03$ mm for anterior–posterior ($n=509$), $24.16\pm 0.97$ mm for superior–inferior ($n=510$), and $24.26\pm 0.96$ mm for medial–lateral ($n=518$) diameters. To determine an inner diameter of the bowl that would accommodate the vast majority of human eyes, we take the measurements in the largest direction (i.e., anterior–posterior) and estimate the 99.85th percentile by assuming that the measurements are from a normal distribution, calculating $\mu +3\sigma =27.53$ mm.

Bartholomew et al.^26^ reported mean, minimum, and maximum values for medial–lateral, superior–inferior, and anterior–posterior diameters for five young pig eyes, 25 young-adult pig eyes, and two older-adult pig eyes. The eyes from older-adult pigs were substantially larger than those from the other pigs. The two older-adult pig eyes were larger in the medial–lateral direction, measuring 29.12 mm and 29.36 mm. To collect more data on adult-pig eyes, we obtained and measured 13 eyes from pigs 1.5–5 years old. For each eye, we measured its largest dimension using digital calipers with 0.01 mm resolution and calculated a mean and standard deviation of $29.42\pm 0.65$ mm. From these, we estimate the 99.85th percentile for adult-pig eyes to be 31.37 mm.

Mohammadi et al.^27^ reported mean diameters of 25.9 mm in the equatorial direction and 25.8 mm in the anterior–posterior direction for sheep eyes, but did not provide any maxima or measures of variance.

Based on the above values, we selected an inner bowl diameter of 32 mm; this will accommodate the 99.85th percentile of adult-pig eyes and also leave room for a layer of masking tape, which is approximately 0.15 mm thick. Because the measurements of pig eyes were substantially larger than the human-eye measurements provided by Augusteyn et al.^25^, we anticipate that human eyes will always fit inside of the bowl, which is fortunate considering that they are costly to obtain. Although we do not have a measure of the 99.85th percentile for sheep-eye diameters, we anticipate that the vast majority of sheep eyes, which are more readily available than pig eyes in some parts of the world (e.g., in the Middle East^27^), will fit inside of the bowl.

### Using a pendulum to approximate an eye’s rotational stiffness

The extraocular muscles and other tissues surrounding the human eye cause viscoelastic behavior when the eye is rotated during surgery. The forces between the surgical instruments and the trocar cannulas are directly related to the orientation of the eye through the eye’s rotational stiffness, so an eye model that has a rotational stiffness similar to the eye of a patient undergoing eye surgery will serve as a more realistic tool for simulating eye surgery. Patients undergoing retinal surgery typically receive a nerve-block anesthetic. Although we do not have measurements for the rotational stiffness of an eye that is affected by a nerve block, we estimate that it will be similar to that of a patient under deep general anesthesia. The force required to rotate the eye of a patient under deep-surgical-plane anesthesia varies approximately linearly with the angle of rotation away from the eye’s primary orientation (assuming the force is applied tangential to the surface of the eye), and has been measured to be 0.3–0.4 gf (i.e., 2.9–3.9 mN) per degree of rotation^18^. Taking the radius of the eye to be 12 mm, this range of forces results in torques ranging from 35 to 47 mN mm per degree of rotation. Converting from degrees to radians, this corresponds to torsional spring constants in the range 2.0–2.7 N mm/rad.

Similar to Mohammadi et al.^24^, we use a pendulum model to approximate the rotational stiffness of an anesthetized eye. Although the gravity-induced torque of a pendulum varies nonlinearly with angular displacement, the torque–displacement relationship approximates the constant stiffness of an anesthetized patient’s eye for the range of eye rotations typical of retinal surgery (i.e., $\le \text {30}^{\circ }$ from the eye’s primary orientation).

**Figure 2:**
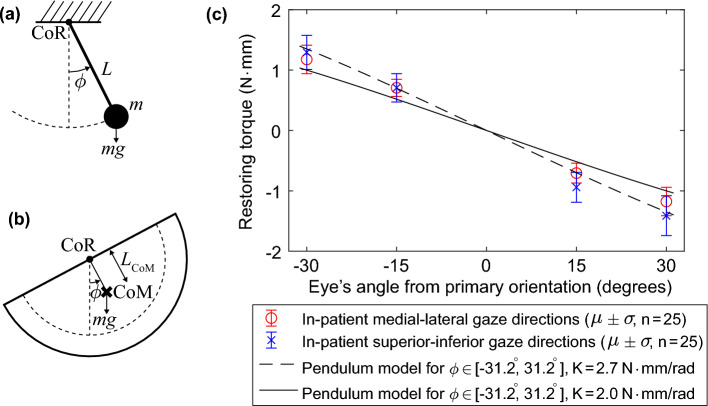
Achieving rotational stiffness. (**a**) A simple pendulum model is used to illustrate the equivalent model for (**b**) the moment about the center of the bowl due to gravity, where the force of gravity acting at the center of mass (CoM) of the bowl creates a moment about the center of rotation (CoR). (**c**) Ranges of restoring torques corresponding to the measurements provided by Rosenbaum and Myer^18^, as well as the torques resulting from the pendulum model at two nominal stiffnesses.

Figure 2 shows how the quasistatic dynamics of the bowl can be approximated as a simple pendulum model, where the center of rotation (CoR) is coincident with the center of the bounding sphere of the bowl. The mass *m* (units kg) of the bowl can be modeled as a point mass at the location of the center of mass (CoM), which is located at a distance $L_{\text {CoM}}$ (units m) from the CoR (Fig. 2b). The acceleration of gravity $g=9.81$ m/s$^2$, which is assumed to be downward, creates a restoring torque

1 $$\begin{aligned} \tau = mgL_{\text {CoM}}\sin \phi \approx mgL_{\text {CoM}}\phi \end{aligned}$$

where $\phi$ is the positive angle measuring the rotation of the eye away from its primary orientation, and the approximate equality in Eq. (1) uses the small-angle approximation $\sin \phi \approx \phi$. This approximates the torque–rotation relationship of a linear torsional spring with a spring constant *K* (units N m/rad), where

2 $$\begin{aligned} K = mgL_{\text {CoM}} \end{aligned}$$

Using the small-angle approximation enables us to compare the approximated spring stiffness *K* of the pendulum with the constants reported in the literature. To compare the true torque–rotation relationships of the pendulum and the eye, which are slightly nonlinear, we directly compare the mean torques measured by Rosenbaum and Myer^18^ and the torques that result from the pendulum model (i.e., using Eq. (1) without the small-angle approximation) for the upper and lower limits of the range of stiffness values described by Rosenbaum and Myer^18^ (i.e., 2.0 N mm/rad and 2.7 N mm/rad). This comparison is illustrated in Fig. 2c.

After a preliminary analysis, we determined that the brim around the edge of the bowl has a negligible effect on the bowl’s rotational stiffness, so we initially design a bowl without a brim, and later verify that the addition of the brim does not have a considerable effect. To approximate a rotational stiffness *K* (units N m/rad) using the bowl, its outer radius *R* (units m) must be

3 $$\begin{aligned} R = \left( \frac{4K}{\pi \rho g} + r^4\right) ^{1/4} \end{aligned}$$

where $\rho$ (units kg/m$^3$) is the density of the material and *r* (units m) is the inner radius of the bowl. The details of this derivation are provided in “Methods” section.

When choosing the rotational stiffness of the device, *K*, we considered the possibility of placing lightweight inserts (e.g., in the form of thin-walled bowls) into the bowl to accommodate eyes with diameters smaller than 32 mm. Because the density of such inserts could be much lower than the density of the solid bowl, the addition of an insert would not significantly increase the rotational stiffness of the device. We chose the rotational stiffness of the device to be 2.0 N mm/rad, which is the lower limit of the range of stiffness values described by Rosenbaum and Myer^18^ and leaves open the possibility of adding a lightweight insert without causing the device to exceed the upper limit of clinically realistic stiffness values (i.e., 2.7 N mm/rad). For the selected value of $K=0.0020$ N m/rad, and $r=0.016$ m chosen previously to accommodate the majority of human and pig eyes, and assuming that the bowl is fabricated using 3D-printed PLA with a measured density of $\rho =1154$ kg/m$^3$, we use Eq. (3) to arrive at $R=0.0232$ m (i.e., $R=23.2$ mm) for the bowl.

In this paper, we use 3D-printed PLA due to its low cost and ease of manufacturing. Although a bowl with $R=23.2$ mm is relatively small, the use of a denser material would result in a smaller bowl, and therefore a more compact device. For example, a bowl made out of 303 stainless steel, with $\rho =8000$ kg/m$^3$, would be required to have $R=17.7$ mm to achieve a stiffness of $K=0.0020$ N m/rad. The use of a denser material would also result in a higher weight of the bowl, making it less likely for the bowl to be inadvertently lifted off of the BTUs. Using Eq. (14), we find that a 303 stainless steel bowl would have a mass of $m=24.2$ g, which is 19% higher than the 3D-printed PLA bowl with a mass of $m=20.3$ g.

### Finalizing BTU placement to enable sufficient eye rotation while minimizing device height

Equipped with the final bowl design (other than the brim), we are able to finalize the locations of the BTUs, and the design of the BTU holder.

**Figure 3:**
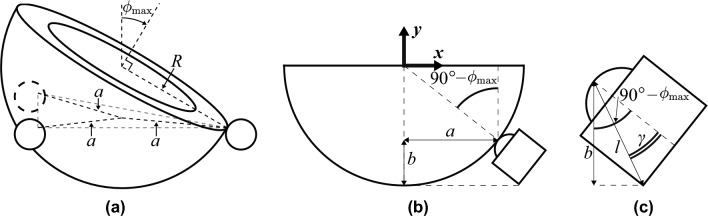
Determining placement of ball transfer units (BTUs). (**a**) Depiction of maximal bowl rotation, at which the edge of the bowl loses contact with a BTU. (**b**) BTU placement that results in the lowest point of the bowl being at the same height as the lowest point on each BTU, with the BTUs placed normal to the bowl’s surface. For clarity, only one BTU is shown. (**c**) Geometric parameters of the BTUs.

The three points of contact between the BTUs and the bowl form an equilateral triangle, with each point a distance *a* from the center of the triangle (Fig. 3a,b). Although the BTU concept is somewhat robust to misalignment, we choose the nominal design to place the BTUs normal to the surface of the spherical bowl (Fig. 3b). For a spherical bowl of outer radius *R*, we will be able to rotate the bowl by an angle

4 $$\begin{aligned} \phi _{\textrm{max}}=90^{\circ }-\sin ^{-1}(a/R) \end{aligned}$$

before the bowl loses contact with one of the BTUs, as depicted in Fig. 3a. Thus, for some desired maximum rotation angle, $\phi _{\textrm{max}}$, we should select

5 $$\begin{aligned} a \le R\sin \left( 90^{\circ } - \phi _{\textrm{max}}\right) \end{aligned}$$

If we would like to provide the most stable (i.e., largest) base possible, then the inequality (5) should be treated as an equality. However, this might result in *a*, and therefore the base of the BTU holder, being so large that it interferes with the volunteer’s nose. On the other hand, as *a* decreases, the overall height of the device increases due to the placement of the BTUs below the bowl.

To limit the height of the device, we consider the value of *a* for which the lowest point on the bowl is at the same height as the lowest points of the BTUs. This configuration is shown in Fig. 3b, with only one BTU shown for clarity. The cross section of the bowl is a semicircle described by

6 $$\begin{aligned} x^2+y^2=R^2 \quad \text {for} \quad y \in [-R,0] \end{aligned}$$

where the coordinate system is at the bowl’s CoR. From the geometry of the BTU (Fig. 3c), we find that

7 $$\begin{aligned} b=l\cos \left( 90^{\circ }-\phi _{\textrm{max}}-\gamma \right) \end{aligned}$$

Referencing Fig. 3b, the point on the semicircle where the BTU contacts the bowl is $(a, {-{(R-b)}})$. Substituting these coordinates into Eq. (6), and combining with Eqs. (4) and (7), we obtain

8 $$\begin{aligned} a^2+\left( R-l\cos \left( \sin ^{-1}\left( \frac{a}{R}\right) -\gamma \right) \right) ^2=R^2 \end{aligned}$$

For our BTUs (Bosch Rexroth R053010810), we measured $l=12.85$ mm and $\gamma =29.2^{\circ }$. We solve Eq. (8) for *a* numerically, choosing the solution in the open interval $\left( 0, R\right)$ where $R=23.2$ mm is the bowl radius that was previously selected to achieve the desired rotational stiffness. This results in $a=19.84$ mm. Substituting this result into Eq. (4), we find that the bowl can be rotated $31.2^{\circ }$ from its upright orientation before losing contact with a BTU, which satisfies our design criterion that the eye should be able to be rotated at least $30^{\circ }$ from its primary orientation.

**Figure 4:**
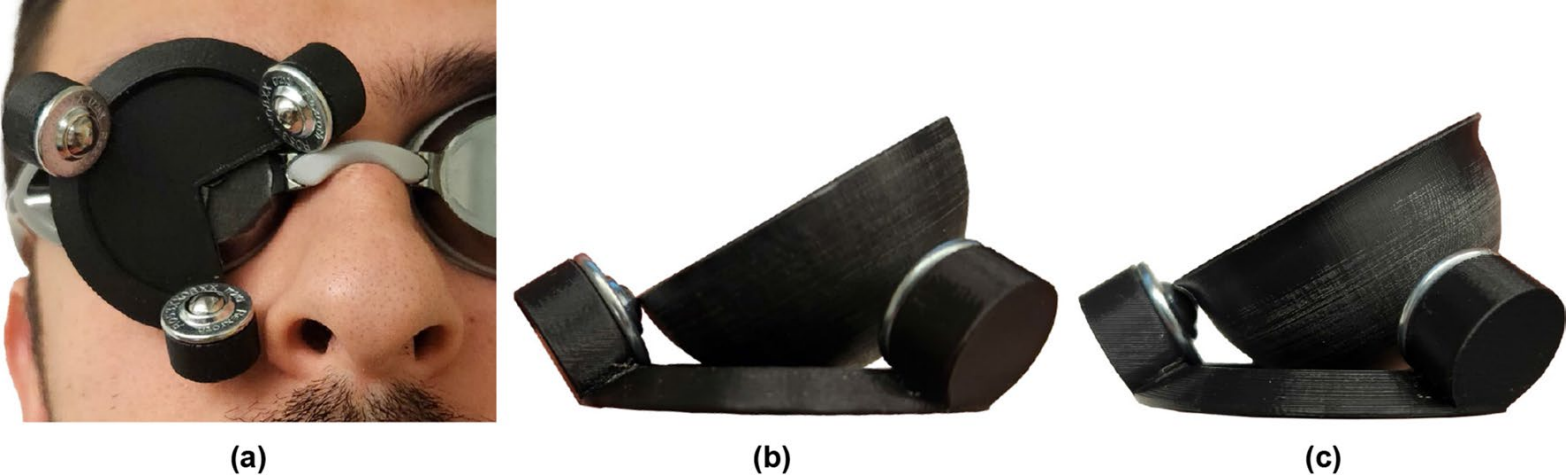
Assembled ball-transfer-unit (BTU) holder and bowl. (**a**) Top view of the BTU holder on swim goggles, with BTUs inserted. A gap in the BTU holder leaves space for the nose and the bridge of the goggles. (**b**) Bowl without a brim, rotated by $\phi _\text {max}$, which is the angle at which it loses contact with a BTU. (**c**) Bowl with a brim, rotated by $\phi _\text {max}$. The brim prevents the bowl from losing contact with the BTUs.

The prototype BTU holder, which places the BTUs in the poses described above, is shown in Fig. 4a. The holder has a section removed to leave space to accommodate the bridge of the volunteer’s nose. The base is (somewhat arbitrarily) 1.5 mm thick and there is a depression to provide (somewhat arbitrary) 0.75 mm of clearance between the bottom of the bowl and the base of the BTU holder.

### Design of the brim for a rotational hard stop

To prevent the bowl from losing contact with the BTUs, we add a brim around the edge of the bowl to act as a rotational hard stop. Without the brim, the bowl may lose contact with a BTU if it is rotated by more than $\phi _\text {max}=31.2^\circ$ (Fig. 4b). We designed the brim such that when the bowl is rotated to its maximum rotation angle, $\phi _{\text {max}}$, the profile of the brim matches that of the BTU and prevents the bowl from rotating farther (Fig. 4c). The brim is (somewhat arbitrarily) 1 mm thick. Using SolidWorks CAD software, we estimate that adding the brim decreases the rotational stiffness of the bowl by only 0.013 N mm/rad, and the stiffness to two significant figures is still 2.0 N mm/rad.

### Evaluation of device by surgeons

**Figure 5:**
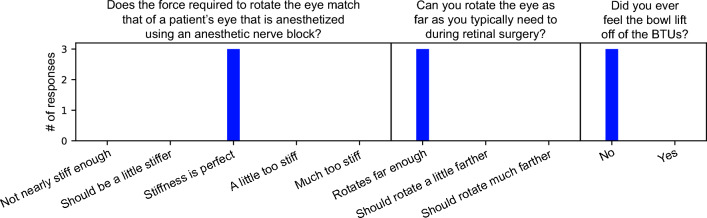
The surgeons’ responses to our questionnaire.

To verify that the rotational stiffness and the range of possible eye orientations are realistic, and that the bowl is not inadvertently lifted off of the BTUs when a mounted eye is rotated, three ophthalmic surgeons used surgical instruments to rotate an enucleated pig eye that was mounted in the final prototype. Trocar cannulas were placed in the eye approximately 3 mm from the limbus at the 4 o’clock and 8 o’clock positions (from the surgeon’s perspective). Instruments were then inserted into the cannulas and used to rotate the eye. The bowl prototype used for initial evaluations had a rough lower surface and did not include the brim. One surgeon indicated that they disliked the feeling due to the rough surface of the bowl, which prompted us to fabricate the bowl as described in “Methods” section. One surgeon indicated that they would prefer that the bowl did not lose contact with the BTUs since the bowl had to be repositioned each time this happened, which motivated the addition of the brim. In later evaluations, the surgeons stated that they liked the addition of the brim. For the final evaluation, each surgeon was given a questionnaire to fill out. The first question asked whether the rotational stiffness of the eye matched that of a patient’s eye that is anesthetized using an anesthetic nerve block (which is typical of retinal surgery), with five possible responses. The second question asked whether the surgeon can rotate the eye as far as they typically need to during retinal surgery, with three possible responses. Finally, the surgeons were asked whether they felt any sensation of the bowl lifting off of the BTUs when they rotated the eye, with two possible responses. The responses from the surgeons were unanimously positive (Fig. 5), so we determined there was no need for further design iteration.

### Maximum rotation of the volunteer’s head

**Figure 6:**
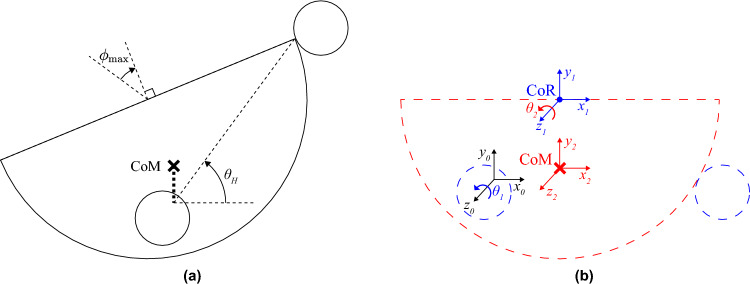
Model used to determine maximum-allowable head rotation. (**a**) The configuration corresponding to the smallest head rotation angle, $\theta _H$, at which the bowl will fall off the ball transfer units (BTUs). The center of mass (CoM) of the bowl is located directly above the line connecting the points at which the two lower BTUs contact the bowl. (**b**) Reference frames used to calculate the rotation of the wearer’s head, $\theta _1$, and the rotation of the bowl, $\theta _2$, at which the bowl will fall off of the BTUs. Each frame shares the same color as the object(s) to which it is rigidly connected.

We assume that the volunteer lies supine, with their head nominally oriented such that the bottom surface of the BTU holder is horizontal. For the BTU poses determined above, we can calculate the smallest angle of rotation of the volunteer’s head, $\theta _H$, at which the bowl will fall off the BTUs, which gives us a measure of stability. Figure 6a shows the configuration that corresponds to the smallest value of $\theta _H$ at which the bowl will fall off. In this worst-case configuration, the bowl is rotated by its maximum angle, $\phi _{\textrm{max}}$, in the direction opposite to the volunteer’s head rotation. To find when the bowl will fall off the BTUs, as a function of both $\theta _H$ and the rotation of the bowl, $\theta _B$, we define coordinate frames that are shown in Fig. 6b. Coordinate Frame 0 is the static world frame, located at the center of the line connecting the points at which the two left BTUs contact the bowl. Coordinate Frame 1 is rigidly connected to the bowl at the CoR, and Coordinate Frame 2 is rigidly connected to the bowl at the CoM. We use kinematics equations to derive the equation describing the configuration at which the bowl falls off of the BTUs as a function of $\theta _B$ and $\theta _H$:

9 $$\begin{aligned} L_{\text {CoM}}\sin \theta _B+\frac{a}{2} = \tan \theta _H\left( \sqrt{R^2-a^2} - L_{\text {CoM}}\cos \theta _B\right) \end{aligned}$$

The details of this derivation are provided in “Methods” section. We substitute the maximum rotation angle of the bowl found previously (i.e., $\theta _B=31.2^{\circ }$) and then solve for $\theta _H$ numerically. We find that the volunteer’s head can rotate (at least) $\theta _H=53.6^{\circ }$ before the bowl falls off the BTUs, suggesting a high degree of stability.

**Figure 7:**
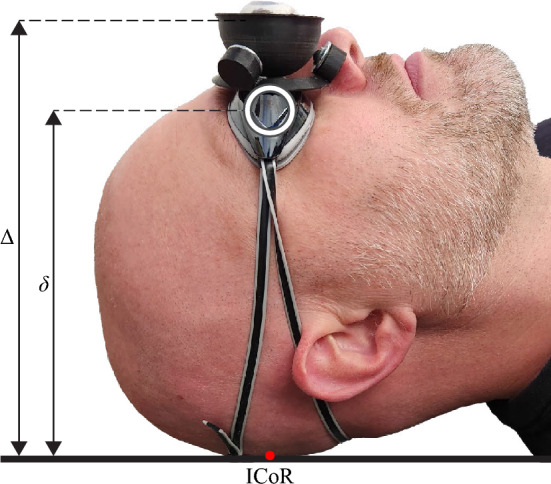
The height of the mounted eye, $\Delta$, above the head’s instantaneous center of rotation (ICoR), will be greater than the height of the volunteer’s eye, $\delta$, which will result in a discrepancy between the movement of the two eyes.

### Effect of the vertical offset of the eye

The offset between the mounted eye and the eye of the volunteer wearing the goggles is expected to result in exaggerated movement (i.e., displacements, velocities, and accelerations) of the mounted eye. Fortunately, this will lead to conservative experiments when evaluating robotic systems, but we would ultimately like the movement of the mounted eye to be as realistic as possible. We can quantify the effect of the offset using a simple model for the motion of the volunteer’s head (Fig. 7). We assume that the volunteer lies supine with their head on a pillow, and as they breathe, their head rolls with a small rotation angle, $\beta$, in the coronal plane. The instantaneous center of rotation (ICoR) of the head is the point at which the head contacts the pillow. Both the eye of the volunteer and the mounted eye are approximately above the ICoR in the nominal configuration. Letting $\delta$ denote the distance from the ICoR to the center of the volunteer’s eye, and $\Delta$ the distance from the ICoR to the center of the mounted eye, we approximate the displacements of the volunteer’s eye and the mounted eye as $\delta \beta$ and $\Delta \beta$, respectively. Thus, the displacement of the mounted eye is greater than that of the volunteer’s eye by a factor of $\Delta /\delta$.

We estimate $\delta$ by combining measurements of the head and eye from the literature with our own measurements, which are all taken in the anterior–posterior direction. We let $l_1$ denote the distance from the back of the head (i.e., the ICoR) to the nasal root depression between the eyes (which is superior to the bridge of the nose), $l_2$ the distance from the nasal root depression to the anterior surface of the eye, and $l_3$ the distance from the anterior surface of the eye to the center of the eye. We can then calculate $\delta$ as

10 $$\begin{aligned} \delta =l_1-l_2-l_3 \end{aligned}$$

To estimate $\Delta$, we let $l_4$ denote the distance from the anterior surface of the eye to the lens of the swim goggles, and let $l_5$ denote the distance from the lens of the swim goggles to the CoR of the mounted eye. We can then calculate $\Delta$ as

11 $$\begin{aligned} \Delta =\delta + l_3 + l_4 + l_5 \end{aligned}$$

To quantify $l_1$, we consider ranges for the distance from the back of the head to the nasal root depression, for males and females, between the 5th and 95th percentiles reported in anthropometry literature^28^; these ranges are $\left[ 185,209\right]$ mm and $\left[ 178,200\right]$ mm for males and females, respectively. To quantify $l_2$, we used SolidWorks CAD software to measure from the nasal root depression to the anterior surface of the eye in the five digital headforms provided by the National Institute for Occupational Safety and Health^29^, which combine measurements from males and females, and we found that the measurements are in the approximate range of $\left[ 11,17\right]$ mm. $l_3$ is the radius of the eye, which is approximately 12 mm^25^. Using digital calipers, we measured $l_4$ to be approximately 11 mm, with little variance, on a few people in our lab. $l_5$ is fully determined by our design parameters, and is 25.5 mm.

For each sex, we numerically search the parameter space of $l_1$ through $l_5$ to find the smallest and largest values for $\Delta /\delta$. The resulting ranges of ratios are $\Delta /\delta \in \left[ 1.26,1.31\right]$ for males, and $\Delta /\delta \in \left[ 1.27,1.33\right]$ for females; however, these ranges only consider error for a given volunteer, which is not particularly meaningful. We can instead consider the ranges for $\delta$, which is proportional to the amount of eye movement, $\delta \beta$, of actual patients, and the ranges for $\Delta$, which is proportional to the amount of movement, $\Delta \beta$, of the mounted eye. We find $\delta \in \left[ 156,186\right]$ mm for males and $\delta \in \left[ 149,177\right]$ mm for females, and $\Delta \in \left[ 205,235\right]$ mm for males and $\Delta \in \left[ 200,226\right]$ mm for females. The ranges do not overlap, meaning that the displacement of the mounted eye will always exceed that of the eyes of actual patients.

## Discussion

Our research group is evaluating the benefits of compensating for patient motion by head-mounting surgical robots^13^. To measure such benefits, our future plans involve performing surgical tasks (e.g., subretinal injections) on enucleated eyes while the robot is mounted to a volunteer’s head. To effectively evaluate the effectiveness of head-mounting, the eye must also be mounted to the volunteer’s head. Our group’s eye-surgery robot^30^ is far too weak to penetrate the goggles in the event of a system failure, and we have already obtained IRB approval for the use of the head-mounted robot and the eye-mounting goggles.

Under some circumstances, it may be necessary to prevent the eye from rotating. For example, if the anterior section of the eye is removed for some experimental reason, then it may not be possible to insert trocar cannulas into the eye, and thus not possible for an instrument to rotate and/or stabilize the eye. For such cases, the bowl can be directly attached to the swim goggles (e.g., using adhesives), since the BTUs and BTU holder are not necessary. For a nonrotating bowl, the brim is not necessary, and the bowl can also have thinner walls to reduce the size of the device.

## Methods

### Choosing the bowl’s outer radius to approximate an eye’s rotational stiffness

The CoM of a solid hemisphere with radius *R* is located at a distance

12 $$\begin{aligned} L_R=\frac{3}{8}R \end{aligned}$$

from the center of its bounding sphere. The volume $V_R$ of such a hemisphere is

13 $$\begin{aligned} V_R=\frac{2}{3}\pi R^3 \end{aligned}$$

and the mass is $m_R = \rho V_R$, where $\rho$ (units kg/m$^3$) is the density of the material. We can model a bowl as a hemisphere of radius *R* with another hemisphere of a smaller radius *r* removed. The result is a bowl of mass

14 $$\begin{aligned} m=\rho \left( V_R-V_r\right) \end{aligned}$$

where $V_r$ is defined analogously to $V_R$. The distance to the CoM of the bowl is given by

15 $$\begin{aligned} L_{\text {CoM}}=\frac{L_R\rho V_R-L_r\rho V_r}{\rho V_R-\rho V_r} = \frac{L_RV_R-L_rV_r}{V_R-V_r} \end{aligned}$$

Combining Eq. (2) and Eqs. (12)–(15), we arrive at the solution for *R*, which is given by Eq. (3).

### Fabrication of final prototype

For our final prototype, depicted in Figs. 1 and 4 (without the bowl), we used Speedo Vanquisher 2.0 swim goggles and Bosch Rexroth R053010810 BTUs. The bowl and BTU holder were both 3D-printed on a Prusa MK3s with a 0.4 mm nozzle diameter, and the filament used was 1.75 mm PLA+ from eSUN. The gcode for the 3D-printer was generated using the software PrusaSlicer. For the bowl, we used slicer software settings for 100% infill, 0.05 mm layer height, and support material. The bowl was printed upside-down on the print plate such that support material was generated inside of the bowl. For the BTU holder, we used settings for 15% infill, 0.2 mm layer height, and support material. The BTU holder was printed such that its surface that attaches to the lens of the swim goggles lay on the print plate. Both STL files are available as supplementary material.

### Maximum rotation of the volunteer’s head

We use the product of exponentials convention for forward kinematics as described by Murray et al.^31^ to derive $g_{02}(\theta _1, \theta _2)$, which is the homogeneous transformation matrix describing the pose of Frame 2 relative to Frame 0 as a function of the angle of the head, $\theta _1=\theta _H$, and the angle of the bowl, $\theta _2=\theta _B$:

16 $$\begin{aligned} g_{02}(\theta _1, \theta _2)=e^{\hat{\xi }_1\theta _1}e^{\hat{\xi }_2\theta _2}g_{02}(0,0) = \begin{bmatrix} R_{02} &{} \varvec{p}_{02} \\ 0 &{} 1 \end{bmatrix} \end{aligned}$$

where $\theta _1\!=\!\theta _2\!=\!0$ corresponds to the reference configuration shown in Fig. 6b. For a rotation of angle $\theta$ about a unit vector $\varvec{\omega }$,

17 $$\begin{aligned} e^{\hat{\xi }\theta }=\begin{bmatrix} e^{\hat{\omega }\theta } &{} (I-e^{\hat{\omega }\theta })(\varvec{\omega } \times \varvec{v}) \\ 0 &{} 1 \end{bmatrix} \end{aligned}$$

where $e^{\hat{\omega }\theta }=I+\hat{\omega }\sin \theta + \hat{\omega }^2(1-\cos \theta )$, $\varvec{v}=-\varvec{\omega } \times \varvec{q}$, $\varvec{q}$ is a vector from the origin of Coordinate Frame 0 to a point on the $\varvec{\omega }$ axis, $\varvec{q}$ and $\varvec{\omega }$ are expressed in Frame 0, and $\hat{\omega }$ is a skew–symmetric matrix containing the components of $\varvec{\omega }$ as

18 $$\begin{aligned} \hat{\omega }=\begin{bmatrix} 0 &{} -\omega _3 &{} \omega _2 \\ \omega _3 &{} 0 &{} -\omega _1 \\ -\omega _2 &{} \omega _1 &{} 0 \end{bmatrix} \end{aligned}$$

For our device, we have

19 $$\begin{aligned} g_{02}(0,0)=\begin{bmatrix} 1 &{} 0 &{} 0 &{} a/2 \\ 0 &{} 1 &{} 0 &{} \sqrt{R^2-a^2}-L_\text {CoM} \\ 0 &{} 0 &{} 1 &{} 0 \\ 0 &{} 0 &{} 0 &{} 1 \end{bmatrix} \end{aligned}$$

and

$$\begin{aligned} \varvec{\omega _1}=\begin{bmatrix}0 \\ 0 \\ 1\end{bmatrix},\, \varvec{q_1}=\begin{bmatrix}0 \\ 0 \\ 0\end{bmatrix},\, \varvec{\omega _2}=\begin{bmatrix}0 \\ 0 \\ 1\end{bmatrix},\, \varvec{q_2}=\begin{bmatrix}a/2 \\ \sqrt{R^2-a^2} \\ 0\end{bmatrix} \end{aligned}$$

The configuration shown in Fig. 6a, where the bowl falls off the BTUs, occurs when $\theta _B=\phi _\text {max}$ and the *x*-component of $\varvec{p}_{02}$ is equal to zero. Using Eq. (16), we find the *x*-component of $\varvec{p}_{02}$, set it equal to zero, and substitute $\theta _1=\theta _H$ and $\theta _2=\theta _B$ to arrive at Eq. (9).

### Supplementary Information

Supplementary Information.

## Data Availability

There is no additional data beyond what has already been provided. All of the .stl files for our device, as fabricated, are included as supplementary material.
